# Evaluation of Chrysanthemi Indici Flos germplasms based on nine bioactive constituents and color parameters

**DOI:** 10.1371/journal.pone.0283498

**Published:** 2023-04-21

**Authors:** Jianling Li, Zi Ye, Min Wei, Changrong Deng, Lianfeng Chi, Lei Xu, Zhengzhou Han, Weifeng Wei

**Affiliations:** 1 State Key Laboratory of Plateau Ecology and Agriculture, Qinghai Plateau Tree Genetics and Breeding Laboratory, Qinghai Academy of Agriculture and Forestry Sciences, Qinghai University, Xining, 810016, China; 2 Resources Sanjiu Medical & Pharmaceutical Co., Ltd., Shenzhen Traditional Chinese Medicine Manufacturing Innovation Center Co., Ltd., China Resources Sanjiu Modern Chinese Medicine Pharmaceutical Co., Ltd., Shenzhen, 518110, China; 3 China Resources Sanjiu (Lu’an) Chinese Traditional Medicine Industry Development Co., Ltd., Lu’an, 237321, China; Foshan University, CHINA

## Abstract

Chrysanthemi Indici Flos (CIF) is the inflorescence of *Chrysanthemum indicum* L., which exists in various shades of yellow and has pharmacologically active constituents. It is widely used for medicinal purposes in China, Japan, and South Korea to treat inflammatory diseases. Its external color is usually used to judge its internal quality in trade; however, the correlation between its color and chemical constituents is unknown. Here, we simultaneously determined five phenylpropanoids (neochlorogenic acid, chlorogenic acid, and isochlorogenic acids A, B, and C) and four flavonoids (linarin, luteolin, apigenin, and acacetin) of 70 CIF germplasms using a newly established UPLC method; furthermore, we measured their color parameters (L*, a*, and b*) using a spectrophotometer. Our results showed considerable variations in the bioactive constituent contents and color parameters of CIF. The content of the five phenylpropanoids and the relative correlation degree *γ*_i_ of the nine constituents were positively correlated with color parameters, which could be rapidly predicted based on L* and/or b*. Moreover, we screened out a high-quality germplasm with a high linarin content and bright colors using the hierarchical clustering method. Our results provide comprehensive insight into CIF’s quality evaluation process, particularly the methods for procuring high-quality medicinal materials and breeding by color.

## Introduction

**Fig 1:**
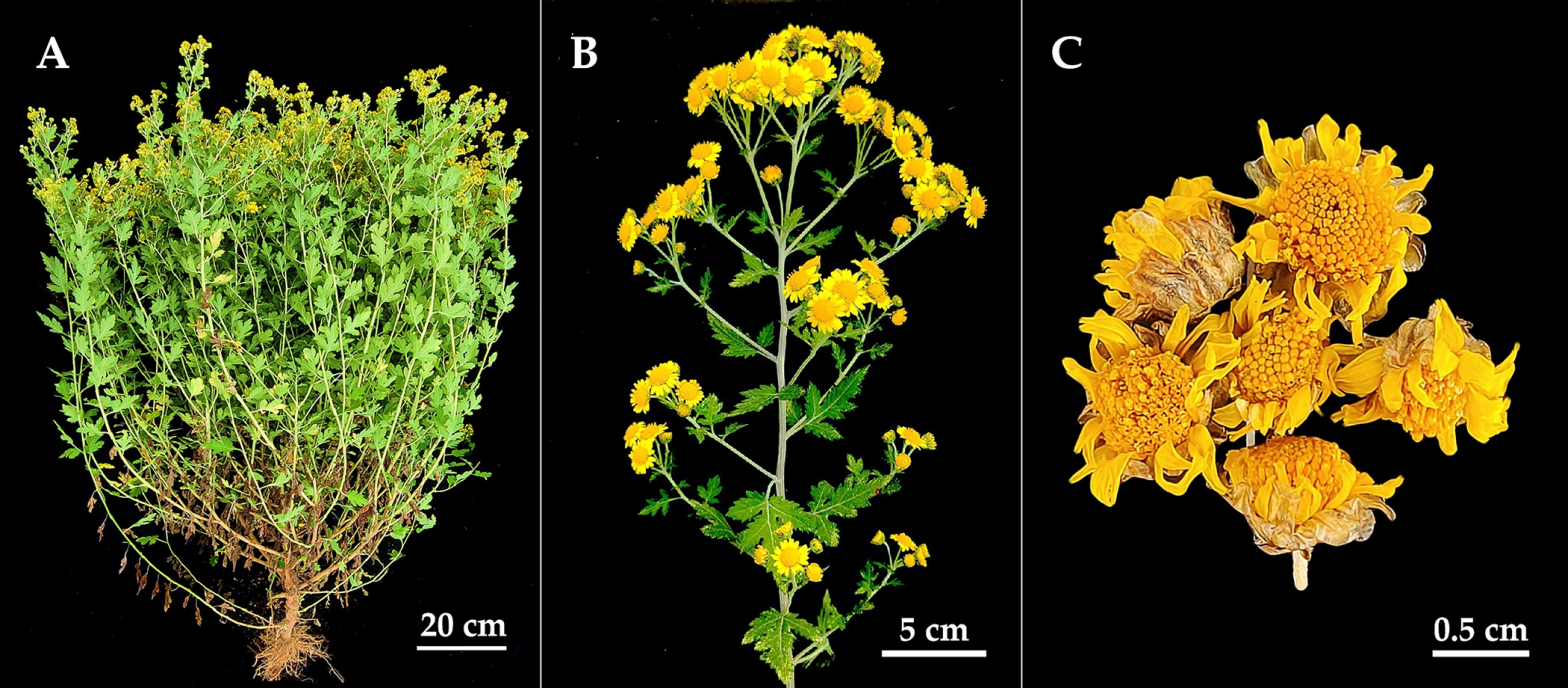
Morphological characteristics of *C*. *indicum* (A) plant, (B) panicles, and (C) dry inflorescence.

Chrysanthemi Indici Flos (CIF), the inflorescence of *Chrysanthemum indicum* L. (Fig 1), has fever-relieving, detoxification effects and possesses anti-inflammatory, hepatoprotective, and antioxidant properties [1,2]. The inflorescence of this plant is used as a material in medicines in Chinese, Japanese, and South Korea pharmacopoeia; furthermore, it was listed as a functional food by the Chinese Ministry of Health in 2012 and is used in 120 kinds of traditional Chinese medicines (TCMs) [3], such as Ganmaoling, Xiasangju and Hepatitis B Qingre Jiedu granules. In China, the annual demand of CIF is as high as 10,000 tons, mainly used for the production of Ganmaoling granule. However, more than 90% CIF is collected from the wild, and its cultivated area is less than 7,000 ha in China [4]. In recent years, due to frequent extreme weather, habitat degradation and over-harvesting, *C*. *indicum* resources have been considerably decreasing, leading to price rises and supply shortages of CIF, which has considerably affected the production of related TCMs [5].

*C*. *indicum*, a polymorphic species with various morphological characteristics and pharmacologically active constituents, is mainly distributed in northeast, southwest, northern, eastern, and central China [6]. In accordance with the *Chinese Pharmacopoeia 2020*, CIF is brownish yellow in color [7]. In CIF production, there are also greenish yellow, pale yellow, yellow, deep yellow, brown, etc. However, bright-colored CIF, such as greenish yellow, yellow, and deep yellow, are favored in medicinal material trade and are usually processed into TCM decoction pieces or tea, while the others are processed into TCMs or dispensing granules.

Medicinal material appearance characteristics, including shape, color and odor, are considerable indicators for quality evaluations, especially the colors that are widely used in trade [8,9]. However, traditional visual identification by eyes is often affected by multiple factors, including the observer and observation conditions. Fortunately, spectrophotometers assist in making quantitative evaluations regarding object color, and they have been used in the analysis of medicinal materials to reveal the relationship between color and chemical constituents, as well as the influence of processing methods on quality, such as for Crataegi Fructus [10], Astragali Radix [11], Schizonepetae Spica [12], etc.

According to the *Chinese Pharmacopoeia 2020*, linarin content, the only chemical marker for CIF quality control, cannot be less than 0.8% [7]; however, more than 70% of CIF from different producing areas of China do not reach this standard [13]. Despite this, CIF quality is often judged by external color in the medicinal material trade for rapid quality assessment. Although it has been demonstrated that drying methods influence CIF color and its chemical constituents [14], the correlation between color and chemical constituents remains unclear. Therefore, it is necessary to reveal the relationship between color and the bioactive constituents of CIF to ensure rapid quality assessments in trade. Moreover, the quality differences between CIFs with different colors for different uses need to be determined.

In our study, we measured CIF color parameters using a spectrophotometer based on the L* a* b* system by the Commission International on Illumination (CIE); nine major bioactive constituents in CIF, including five phenylpropanoids (neochlorogenic acid, chlorogenic acid, and isochlorogenic acids A, B, and C) and four flavonoids (linarin, luteolin, apigenin, and acacetin), were determined simultaneously using a newly established ultra-performance liquid chromatography (UPLC) method; furthermore, grey relational analysis (GRA), a quantitative comparative analysis method that is widely used in the quality assessment of TCMs [15], was performed to evaluate the comprehensive quality of CIF according to the contents of the nine bioactive constituents.

## Materials and methods

### Field trial design

From 2014 to 2020, 70 wild *C*. *indicum* germplasms were collected from 11 provinces in China, including Anhui, Henan and Hubei, the main CIF-producing areas (Table 1). These germplasms were identified by their morphological characteristics according to the description of *Flora of China* [6] and were transplanted to a CIF germplasm resource nursery. The nursery was built at Yangxin county (N 29°55′12″, E 115°3′53″), Hubei province by China Resources Sanjiu Medical & Pharmaceutical Co., Ltd. These germplasms were propagated via shoot cuttings, transplanted to a field with red soil near the nursery in a randomized block design with three replications, and planted in a 0.5 × 0.9 m spacing between plants and rows on May 10, 2020. Field water and fertilizer management were in accordance with the local standard of Hubei province “Chinese medicinal materials—code of practice for Chrysanthemi Indici Flos production” (DB42/T 1768–2021). From late October to late November, we harvested CIF at the early flowering stage (tongue flowers unfolded and tubular flowers partially unfolded), dried at 60°C in an 101–3 oven (Shanghai Guangdi Instrument Equipment Co., Ltd, Shanghai, China) for 16 h, and crushed through a 100-mesh sieve.

**Table 1:** Information for *C*. *indicum* germplasm collection sites.

No. of germplasms	Collection sites		No.of germplasms	Collection sites	
	Province of China	County, City		Province of China	County, City
AHFN	Anhui	Funan, Fuyang	HAX	Henan	Xinxian, Xinyang
AHHN		Huaining, Anqing	HAXC		Xincai, Zhumadian
AHHQ		Huoqiu, Luan	HBBD	Hubei	Badong, Enshi
AHHS		Huoshan, Luan	HBCA		Congyang, Xianyang
AHJZ		Jinzhai, Luan	HBCY		Changyang, Yichang
AHLQ		Linquan, Fuyang	HBDW		Dawu, Xiaogan
AHQS		Qianshan, Anqing	HBDY1		Daye, Huangshi
AHSS		Susong, Anqing	HBDY2		Daye, Huangshi
AHTC1		Tongcheng, Anqing	HBGS		Guangshui, Suizhou
AHTC2		Tongcheng, Anqing	HBHS		Hongshan, Wuhan
AHTH		Taihu, Anqing	HBJL		Jianli, Jingzhou
AHWJ		Wangjiang, Anqing	HBLT1		Luotian, Huanggang
AHGY		Guoyang, Bozhou	HBLT2		Luotian, Huanggang
AHYS		Yingshang, Fuyang	HBLW		Longwan, Qianjiang
AHZY		Zongyang, Tongling	HBMC		Macheng, Huanggang
CQWX	Chongqing	Wuxi	HBQC		Qichun, Huanggang
GDLZ	Guangdong	Lianzhou, Qingyuan	HBTF		Tuanfeng, Huanggang
GDPY		Pingyuan, Meizhou	HBTS		Tongshan, Xianning
GXFC	Guangxi	Fuchuan, Hezhou	HBXS		Xishui, Huanggang
GXGP1		Guiping, Guigang	HBYX		Yangxin, Huangshi
GXGP2		Guiping, Guigang	HNTJ	Hunan	Taojiang, Yiyang
GXQZ		Quanzhou, Guilin	JSXW	Jiangsu	Xuanwu, Nanjing
GXXA		Xingan, Guilin	SCFC	Sichuan	Fucheng, Mianyang
GXYZ		Yizhou, Hechi	SNDF	Shaanxi	Danfeng, Shangluo
GZJS	Guizhou	Jinsha, Bijie	SNHY		Huayin, Weinan
HABY	Henan	Biyang, Zhumadian	SNHZ1		Huazhou, Weinan
HADC		Dancheng, Zhoukou	SNHZ2		Huazhou, Weinan
HAGS		Gushi, Xinyang	SNQ		Qianxian, Xianyang
HAHB		Huaibin, Xinyang	SNSN		Shangnan, Shangluo
HAHC		Huangchuan, Xinyang	SNXX		Xixiang, Hanzhong
HAI1		Xixian, Xinyang	SXRC	Shanxi	Ruicheng, Yuncheng
HAI2		Xixian, Xinyang	SXTG1		Taigu, Jinzhong
HALB		Lingbao, Sanmenxia	SXTG2		Taigu, Jinzhong
HAPY		Pingyu, Zhumadian	SXZZ		Zezhou, Jincheng
HASC		Shangcai, Zhumadian	YNJC	Yunnan	Jiangchuan, Yuxi

### Extraction of the bioactive constituents from CIF

Based on the methods of the *Chinese Pharmacopoeia 2020*, accurately weighed CIF powder (0.250 g) was transferred into a conical flask with 100 ml 70% methanol aqueous solution and extracted by refluxing for 3 hours. Then, we cooled the extraction to room temperature, compensated the loss weight with 70% methanol aqueous solution and filtered the supernatant using a 0.22 um millipore filter. We stored the subsequent filtrate at 4°C until UPLC analysis.

### Preparation of mixed standard solution

Reference standards (Table 2) were weighed accurately, transferred into a brown volumetric flask, suspended in 70% methanol aqueous solution and then stored at 4°C. We filtered the mixed reference solution using a 0.22 um millipore filter until UPLC analysis. There were 21.23 μg·mL^-1^ neochlorogenic acid, 21.19 μg·mL^-1^ chlorogenic acid, 22.44 μg·mL^-1^ isochlorogenic acid B, 22.81 μg·mL^-1^ isochlorogenic acid A, 20.17 μg·mL^-1^ isochlorogenic acid C, 45.90 μg·mL^-1^ linarin, 20.70 μg·mL^-1^ luteolin, 24.10 μg·mL^-1^ apigenin, and 21.81 μg·mL^-1^ acacetin in the prepared mixed reference solution.

**Table 2:** Manufacturer and batch number of reference standards.

Reference standards	Manufacturers	Purity	Batch number
Acacetin	Shanghai Hongyong Biotechnology Co., Ltd, Shanghai, China	≥ 98.0%	100021–201910
Neochlorogenic acid	Shanghai Yuanye Biotechnology Co., Ltd, Shanghai, China	≥ 98.0%	M07GB140938
Isochlorogenic acid B		≥ 98.0%	P17N11L131404
Isochlorogenic acid C		≥ 98.0%	M07GB140939
Chlorogenic acid	National Institutes for Food and Drug Control, Beijing, China	≥ 96.1%	110753–202018
Isochlorogenic acid A		≥ 94.3%	111782–201807
Linarin		≥ 98.0%	111528–202112
Luteolin		≥ 94.4%	111520–202006
Apigenin		≥ 99.4%	111901–202004

### UPLC conditions

We conducted identification and quantification of the nine bioactive constituents on a Waters Acquity UPLC^TM^ system H-CLASS (Waters Corp., Milford, MA, USA), which was equipped with a binary solvent delivery manager and a sample manager. We performed chromatographic separations using an Acquity UPLC HSS C_18_ column (150 × 3.0 mm, 1.8 μm, Waters Corp., Milford, MA, USA). The mobile phase was composed of solvents A (100% acetonitrile) (Thermo Fisher, San Jose, CA, USA) and B (0.05% formic acid aqueous solutions) (Guangdong Guanghua Sci-Tech Co., Ltd., Shantou, Guangdong, China). We maintained the column temperatures, flow rates, injection volumes of the reference standards and samples, and detection wave lengths at 25°C, 0.4 mL·min^-1^, 3.0 μL, and 326 nm, respectively. We optimized the gradient program as follows: 0–6 min, 10%-18% A; 6–7 min, 18%-20% A; 7–12 min, 20%-20% A; 12–19 min, 20%-23% A; 19–20 min, 23%-28%; 20–25 min, 28%-30% A; 25–38 min, 30%-40% A; 38–40 min, 40%-65% A; 40–42 min, 65%-90% A; 42–44 min, 90%-90% A; 44–44.1 min, 90%-10% A; 44.1–47 min, 10%-10% A.

### Color measurement

We transferred the CIF powder of the 70 *C*. *indicum* germplasms into a glass cell (ф 4.8 cm, Shenzhen Three NH Technology Co., Ltd., Shenzhen, Guangdong, China) and measured the color parameters (lightness-shade chromaticity L*, red-green chromaticity a*, yellow-blue chromaticity b*) using a YS3060 spectrophotometer (Shenzhen Three NH Technology Co., Ltd., Shenzhen, Guangdong, China) with a pulsed xenon arc lamp (D65, ф 8mm, 400–700 nm) and a 10° observer. We measured each CIF sample 3 times. Additionally, we calibrated the spectrophotometer by black and white correction plate before use. Moreover, we determined the standard color difference value E*_ab_ using the formula:

$$E_{ab}^{*}=\sqrt{{\left(L^{*}\right)}^{2}+{\left(a^{*}\right)}^{2}+{\left(b^{*}\right)}^{2}}$$

### Data analysis

We used MS Excel 2019 (Microsoft, Redmond, WA, USA) to calculate the relative correlation degree *γ*_i_ of the nine bioactive constituents of CIF based on the method outlined by Hua et al. [15] to evaluate its comprehensive quality. The Shannon’s Diversity Index *H*’ of the bioactive constituents, *γ*_i_ and the color parameters (L*, a* and b*) of CIF were calculated using the PCH’ Diversity Index Calculation Tool designed by the researcher Beiluozhongyuan (https://pan.baidu.com/s/1jG3eWKI). We conducted Pearson correlation and multiple linear regression (MLR) analyses using SPSS v21 (IBM, Chicago, IL, USA) to elucidate the relationships between the nine bioactive constituents, *γ*_i_ and their color parameters. We performed cluster analysis on the *γ*_i_ and color parameters using TBtools v1.0983 [16] and SPSS v21 with the hierarchical clustering method to create a clustering heat map and to calculate rescaled distance cluster combinations, respectively. We analyzed significant differences between the *γ*_i_ and color parameters among different cluster groups using SPSS v21 using one-way ANOVA followed by Fisher’s least significant difference (LSD) test.

## Results

### Methodological investigation

**Fig 2:**
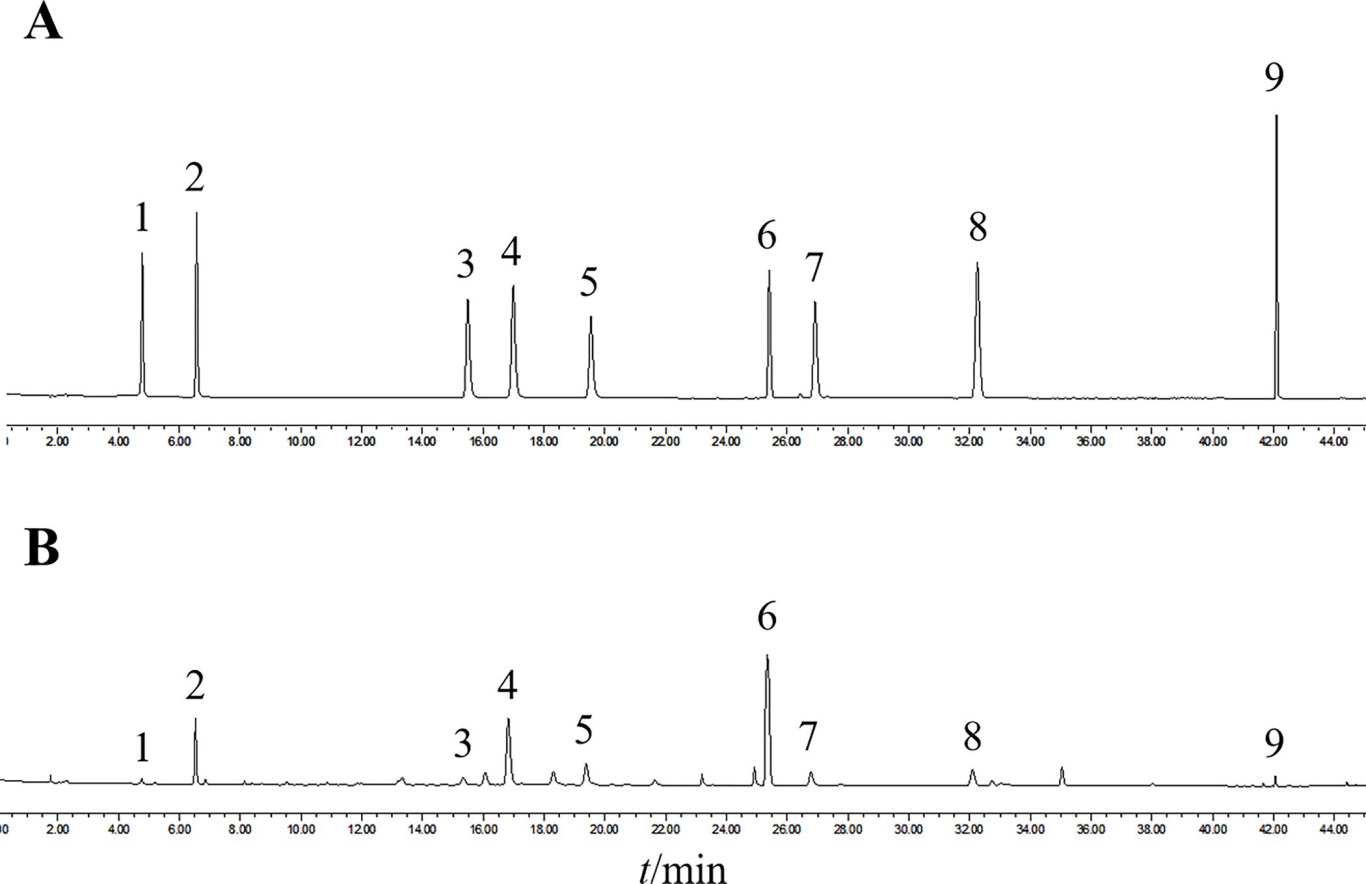
UPLC chromatograms of (A) the mixed reference solutions and (B) a CIF sample: 1, neochlorogenic acid; 2, chlorogenic acid; 3, isochlorogenic acid B; 4, isochlorogenic acid A; 5, isochlorogenic acid C; 6, linarin; 7, luteolin; 8, apigenin; 9, acacetin.

**Analytical method validation.** The CIF sample extractions and the mixed reference solutions were detected according to the above-mentioned UPLC conditions. The chromatographic resolution between component peaks was greater than 1.5, and the number of theoretical plates was more than 31,000. The nine components in the mixed reference solution and the CIF sample extractions showed high-resolution separation without interference, and their retention time was stable (Fig 2).

#### Linearity

To assess the linear range of the nine constituents, we diluted the mixed reference solutions with 70% methanol by 2, 4, 8, 16, 32, and 64 times, and detected the constituents according to the above-mentioned UPLC conditions. We drew the standard curve with the peak area as the ordinate and the concentration of the reference solution as the abscissa. The calibration curves of nine bioactive constituents had good linearity in their concentration ranges and the *R*^2^ values were all higher than 0.999 (Table 3 and S1 Fig).

**Table 3:** Linearity assessment of the nine reference standards under UPLC conditions.

**Reference standards**	**Regression equation**	**Range (μg·mL** ^**-1**^ **)**	***R*2**
Neochlorogenic acid	y = 19972x - 1426.8	0.332–21.230	0.9999
Chlorogenic acid	y = 20513x - 1040.5	0.342–21.910	1.0000
Isochlorogenic acid B	y = 18849x - 4151.9	0.351–22.440	0.9997
Isochlorogenic acid A	y = 25667x - 6403.4	0.356–22.810	0.9996
Isochlorogenic acid C	y = 23658x - 4693.5	0.315–20.170	0.9997
Linarin	y = 16494x + 20.799	0.717–45.900	1.0000
Luteolin	y = 21640x - 3773.5	0.323–20.700	0.9998
Apigenin	y = 32759x - 4044.2	0.377–24.100	1.0000
Acacetin	y = 30240x - 1191.3	0.341–21.810	1.0000

#### Precision

We accurately weighed a sample of CIF powder six times to ensure that the same weight was obtained, extracted the bioactive constituents from CIF and detected the constituents according to the above-mentioned UPLC conditions. The RSD was less than 2% (S1 Table).

#### Stability

Sample extraction was determined according to the above-mentioned UPLC conditions at 0, 2, 4, 8, 16, 24, 36, and 48 h after preparation. The RSD of the nine bioactive constituent peak areas at different times was less than 2% (S2 Table).

#### Recovery

We accurately weighed a detected constituents in the CIF powder (0.125 g) six times, added reference solutions, extracted the bioactive constituents and detected constituents according to the above-mentioned UPLC conditions. The recovery and the RSD of the nine bioactive constituents was between 98.62% and 101.57%, and between 0.31% and 1.69%, respectively (S3 Table).

### Genetic diversity analysis of CIF bioactive constituents, *γ*_i_ and color parameters

The bioactive constituents, *γ*_i_ and color parameters of *C*. *indicum* germplasms all exhibited abundant genetic variation (*H*’ > 3) (Table 4). The mean concentration of the nine bioactive constituents of CIF followed this order: linarin > isochlorogenic acid A > chlorogenic acid > isochlorogenic acid C > luteolin > isochlorogenic acid B > apigenin > acacetin > neochlorogenic acid. According to the *Chinese Pharmacopoeia 2020*, only 41.428% of CIF germplasm linarin content reached 0.8% and qualified. Among these bioactive constituents, linarin had the highest *CV* (120.564%) and the smallest *H*’ (3.456). In contrast, neochlorogenic acid had the highest *H*’ (4.106) and the smallest *CV* (54.938%). The values of L*, a* and b* were 53.938–71.312, -2.620–5.240, and 18.240–39.397, respectively. Therefore, the predominant CIF color was white, its subordinate color was yellow, and E*_ab_ was mainly affected by L* and b*.

**Table 4:** Genetic diversity analysis of constituents, *γ*_i_, and color parameters of CIF.

Phenotypes	Max.	Min.	Mean	SD	*CV* (%)	*H*’
Neochlorogenic acid	0.053	0.004	0.018	0.010	54.938	4.106
Chlorogenic acid	0.815	0.052	0.282	0.178	63.253	4.067
Isochlorogenic acid B	0.574	0.007	0.113	0.121	106.702	3.825
Isochlorogenic acid A	1.888	0.007	0.427	0.399	93.384	3.887
Isochlorogenic acid C	0.429	0.006	0.171	0.098	57.686	4.075
Linarin	3.284	0.001	0.935	1.127	120.564	3.456
Luteolin	0.403	0.010	0.138	0.084	60.991	4.073
Apigenin	0.152	0.003	0.033	0.027	82.264	3.959
Acacetin	0.145	0.000	0.020	0.024	120.246	3.701
*γ*_i_	0.656	0.375	0.480	0.056	11.604	4.242
L*	71.312	53.938	62.507	3.857	6.171	4.247
*a* ^*^	5.240	-2.620	2.246	1.588	70.712	4.098
*b* ^*^	39.397	18.240	27.202	5.121	18.826	4.232
Color simulation and hexadecimal codes	#C9AA66	#858261	#A89567			

### Correlation analysis of CIF bioactive constituents and color parameters

CIF’s bioactive constituent concentration was correlated with L*, a* and b* values (Table 5). Both neochlorogenic acid and chlorogenic acid were significantly positively correlated with L* (*P* = 0.028 and 0.002, respectively) and b* (*P* = 0.011 and 0.023, respectively). The a* value was significantly negatively correlated with chlorogenic acid (*P* = 0.016), but had no significant correlation with neochlorogenic acid (*P* = 0.260). Isochlorogenic acid A and *γ*_i_ were significantly positively correlated with L* value (*P* = 0.007 and 0.045, respectively). Isochlorogenic acid C and acacetin were significantly positively (*P* = 0.049) and negatively (*P* = 0.027) correlated with b*, respectively. However, linarin, luteolin, and apigenin had no significant correlation with its color parameters (Table 5).

**Table 5:** Correlation analysis of CIF constituents, *γ*_i_ and color parameters.

Constituents and *γ*_i_	L*		a*		b*	
	*r*	*P*-value	*r*	*P-*value	*r*	*P*-value
Neochlorogenic acid	0.264	0.028	-0.137	0.260	0.301	0.011
Chlorogenic acid	0.358	0.002	-0.287	0.016	0.271	0.023
Isochlorogenic acid B	0.169	0.162	-0.079	0.516	0.266	0.026
Isochlorogenic acid A	0.319	0.007	-0.199	0.098	0.207	0.085
Isochlorogenic acid C	0.177	0.142	-0.126	0.299	0.237	0.049
Linarin	0.100	0.396	-0.088	0.466	-0.076	0.533
Luteolin	-0.048	0.695	0.076	0.531	0.084	0.49
Apigenin	-0.018	0.885	0.028	0.819	-0.092	0.448
Acacetin	-0.131	0.28	0.007	0.956	-0.264	0.027
*γ*_i_	0.240	0.045	-0.160	0.185	0.192	0.111

### Multiple linear regression analysis on CIF bioactive constituents, *γ*_i_ and color parameters

The concentrations of neochlorogenic acid (corrected *R*^2^ = 0.077, *P* = 0.011), chlorogenic acid (corrected *R*^2^ = 0.115, *P* = 0.002), isochlorogenic acid B (corrected *R*^2^ = 0.057, *P* = 0.026), isochlorogenic acid A (corrected *R*^2^ = 0.089, *P* = 0.007), isochlorogenic acid C (corrected *R*^2^ = 0.042, *P* = 0.049), acacetin (corrected *R*^2^ = 0.056, *P* = 0.027) and *γ*_i_ (corrected *R*^2^ = 0.044, *P* = 0.045) had linear relationships with CIF’s color parameters (Tables 6 and 7). However, linarin, the chemical marker of CIF in *Chinese Pharmacopoeia 2020*, as well as other constituents, were lacking a linear relationship with CIF color parameters. We established multiple linear regression equations for estimating the concentrations of neochlorogenic acid, chlorogenic acid, isochlorogenic acids A, B, and C, acacetin, and *γ*_i_ in CIF, as follows:

$$Neochlorogenic\;acid\;\%=0.001\;b^{*}$$ (1)

$$Chlorogenic\;acid\;\%=0.017\;L^{*}-0.751$$ (2)

$$Ischlorogenic\;acid\;A\;\%=0.033\;L^{*}-1.636$$ (3)

$$Isochlorogenic\;acid\;B\;\%=0.006\;b^{*}$$ (4)

$$Ischlorogenic\;acid\;C\;\%=0.005\;b^{*}$$ (5)

$$Acacetin\;\%=-0.001\;b^{*}+0.055$$ (6)

$$\gamma_{i}\%=0.003\;L^{*}+0.263$$ (7)

**Table 6:** Multiple regression analysis on the constituents, *γ*_i_, and color parameters of CIF.

Dependent variables	*R*	*R* ^2^	Corrected *R*^2^	Estimated *SE*
Neochlorogenic acid	0.301	0.091	0.077	0.009
Chlorogenic acid	0.358	0.128	0.115	0.168
Isochlorogenic acid B	0.266	0.071	0.057	0.117
Isochlorogenic acid A	0.319^a^	0.102	0.089	0.381
Isochlorogenic acid C	0.237	0.056	0.042	0.096
Linarin	0.256	0.065	0.023	1.114
Luteolin	0.182	0.033	-0.011	0.085
Apigenin	0.134	0.018	-0.027	0.028
Acacetin	0.264	0.070	0.056	0.024
*γ*_i_	0.240	0.058	0.044	0.054

**Table 7:** ANOVA analysis on the constituents, *γ*_i_, and color parameters of CIF.

Dependent variables	Model	*Sum of Squares*	*Mean Square*	*F*	*P*
Neochlorogenic acid	Regression	0.001	0.001	6.777	0.011
	Residual	0.006	0.000		
	Total	0.007			
Chlorogenic acid	Regression	0.280	0.280	9.976	0.002
	Residual	1.910	0.028		
	Total	2.190			
Isochlorogenic acid B	Regression	0.071	0.071	5.175	0.026
	Residual	0.935	0.014		
	Total	1.006			
Isochlorogenic acid A	Regression	1.118	1.118	7.718	0.007
	Residual	9.847	0.145		
	Total	10.964			
Isochlorogenic acid C	Regression	0.037	0.037	4.034	0.049
	Residual	0.631	0.009		
	Total	0.669			
Linarin	Regression	5.740	1.913	1.541	0.212
	Residual	81.971	1.242		
	Total	87.711			
Luteolin	Regression	0.016	0.005	0.755	0.523
	Residual	0.474	0.007		
	Total	0.491			
Apigenin	Regression	0.001	0.000	0.403	0.752
	Residual	0.051	0.001		
	Total	0.052			
Acacetin	Regression	0.003	0.003	5.090	0.027
	Residual	0.038	0.001		
	Total	0.041			
*γ*_i_	Regression	0.012	0.012	4.162	0.045
	Residual	0.201	0.003		
	Total	0.214			

### Custer analysis of *γ*_i_ and color parameters

**Fig 3:**
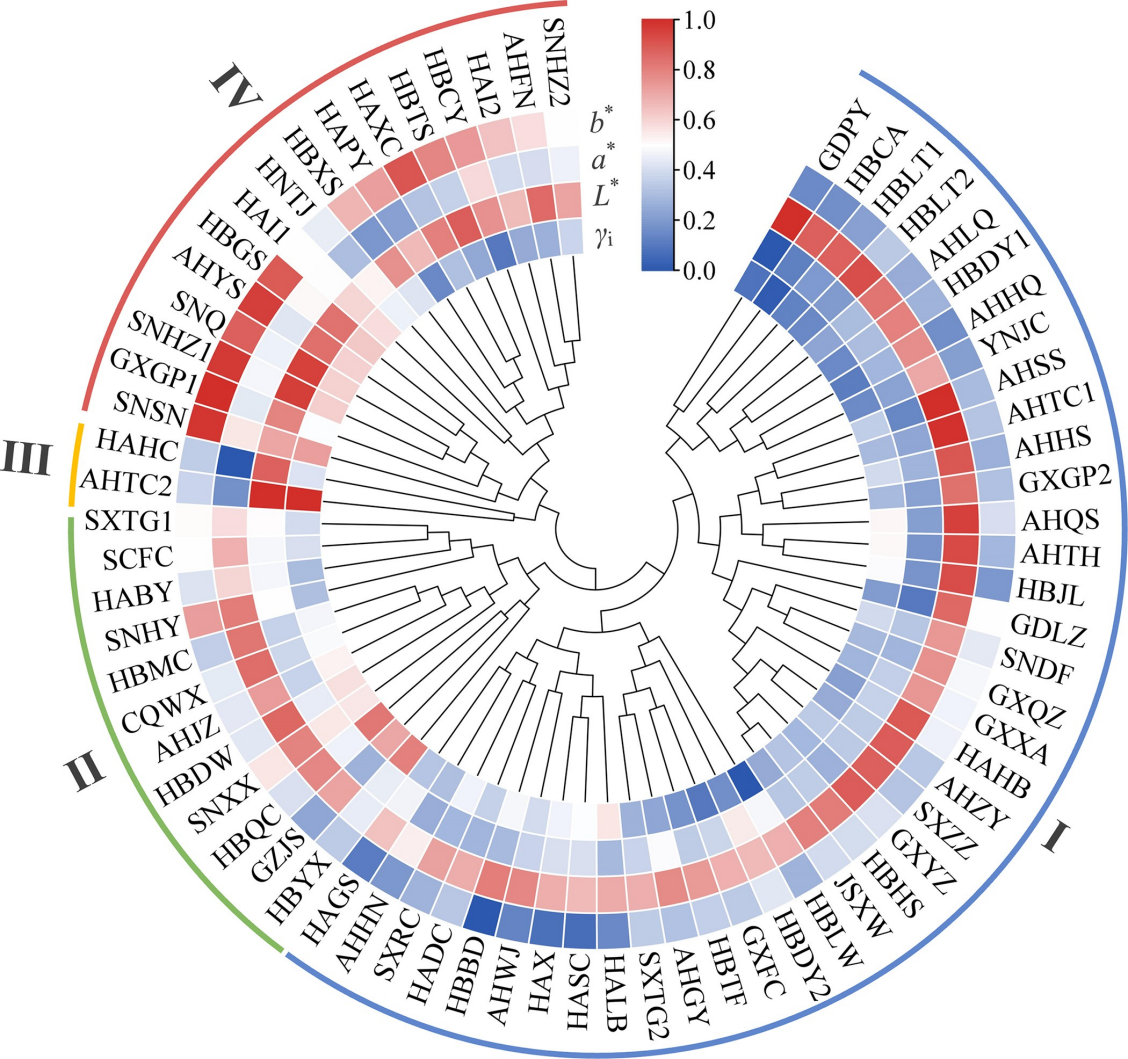
Cluster analysis heat map of *γ*_i_ and color parameters of CIF.

When the rescaled distance cluster combination was 15, the 70 *C*. *indicum* germplasms were divided into four groups (Figs 3 and S2 and Table 8). The 40 germplasms in Group Ⅰ had the highest a*, and the lowest *γ*_i_, L*, b*, and E*_ab_. Therefore, these germplasms were of poor quality and had a dingy yellow color. The 12 germplasms in Group Ⅱ had higher *γ*_i_, L*, b* and E*_ab_, and lower a* compared with Group Ⅰ, and their quality and color were also superior. Group Ⅲ contained only two germplasms (AHTC2 and HAHC), and they had the highest *γ*_i_ and L*, and the lowest a*; therefore, they had the highest quality and the most brightness compared with the others. As the *γ*_i_, L*, b*, and linarin of AHTC2 were higher than those of HAHC, AHTC2 was more suitable for medicine or tea. Sixteen germplasms in Group Ⅳ had the highest b* and E*_ab_, although we observed no considerable differences in their comprehensive quality *γ*_i_ with Group Ⅱ.

**Table 8:** Inter-comparison of *γ*_i_, L*, a*, and b* in different groups (mean ± SD).

Groups	*γ*_i_	L*	a*	b*	E*_ab_	Color simulation and hexadecimal codes
Ⅰ	0.455±0.040^Bc^	60.380±1.718^Cd^	3.015±0.773^Aa^	24.324±2.606^Cd^	65.216±1.888^Cc^	#A68E67
Ⅱ	0.522±0.048^Aab^	62.586±1.202^Bc^	2.461±0.892^Ab^	27.713±2.589^Bbc^	68.524±2.012^Bb^	#AC9466
Ⅲ	0.572±0.114^Aa^	70.357±1.351^Aa^	-2.019±0.850^Cd^	25.932±0.373^BCcd^	75.014±1.373^Aa^	#BAAC7D
Ⅳ	0.493±0.053^ABb^	67.748±1.888^Ab^	0.287±0.787^Bc^	34.378±3.991^Aa^	76.035±3.149^Aa^	#BAA367

Different lowercase and uppercase letters indicated significant differences at 0.01 < *P* < 0.05 and *P* < 0.001, respectively; same lowercase and uppercase letters indicated no significant differences at *P* > 0.05 and *P* > 0.001, respectively.

## Discussion

Medicinal materials have many kinds of pharmacology constituents that play considerable roles in physiological regulation through synergistic or antagonistic effects, and it is inappropriate to rely on only a few constituents to assess their quality [17,18]. GRA is a method used for the comprehensive description and quantitative evaluation of multiple factors, and makes full use of the information differences of related things to compare and sort complex systems, as well as to realize dimensions and simplification reductions of information [19,20]. It has been used to evaluate the quality of medicinal materials, such as Pseudostellariae Radix [15], Magnoliae Officinalis Cortex [21], Prunellae Spica [22], etc. In recent years, large amounts of bioactive constituents with various pharmacological effects have been found in CIF, including flavonoids, phenylpropanoids, terpenoids, etc. [1,2]. In our study, we used GRA to comprehensively assess the quality of 70 CIF germplasms by combing nine bioactive constituents instead of only relying on linarin. Our results showed that there were rich genetic diversities in the bioactive constituent content, *γ*_i_, and the color of *C*. *indicum* germplasms, and that there were considerable correlations among them. We could quickly predict the *γ*_i_ and phenylpropanoid content of CIF based on its color. However, we found no significant correlation between the CIF color and its chemical maker linarin. Therefore, CIF’s external color can be used to evaluate its internal quality in the medicinal material trade and for breeding; however, we cannot assess whether its linarin content conforms to the standard of the *Chinese Pharmacopoeia 2020* through its color.

Our results showed that the bioactive constituent content and the color parameters of the 70 CIF germplasms were different compared with each other; these germplasms were clustered into different groups. For example, we clustered 16 germplasms from 13 prefecture-level cities of seven provinces of China into Group Ⅳ. Moreover, the linarin content of these germplasms, which comprised more than 58%, was not up to *Chinese Pharmacopoeia 2020* standard. These CIF characteristics, including rich genetic diversity and low linarin content, have been reported in several studies. It has been reported that there was high genetic diversity among *C*. *indicum* geographic populations, and their genetic distances showed no considerable correlations with geographic distance [23]. Furthermore, the contents of bioactive constituents (chlorogenic acid, galuteolin, isochlorogenic acids A, B and C, and linarin) of CIF samples from different provinces of China differed greatly from each other, especially for linarin; only 36.4% of the sample linarin content was up to the standard of the *Chinese Pharmacopoeia 2020* [24]. Although our study results showed that the constituent contents of CIF germplasms from different geographical areas varied greatly, similar to other medicinal crops, this character was mainly determined by their genetic characteristics, followed by altitude, temperature, precipitation and other external environmental factors [25,26]. For example, natural hybrid sexual and asexual propagation materials from the CIF cultivar "999YEJU1", with high levels of linarin (>2.5%) bred by China Resources Sanjiu Medical & Pharmaceutical Co., Ltd., were planted in different provinces of China, including Anhui, Henan, Hebei, Guangxi, Hunan, etc.; the CIF linarin content still remained at a high level (>2.5%) [27].

*C*. *indicum* is a cross-pollination plant with maternal inheritance, and its phenotypes, including morphological character and chemical components of its sexual offspring, are partially similar to its maternal parent [28,29]. In our study, we screened one germplasm AHTC2 with bright color and high quality, which was suitable as a material for TCMs, decoction pieces, dispensing granules or tea drinks. Similar to other species of *Chrysanthemum*, our selected germplasm can be expanded by asexual propagation to realize popularization; however, the cost is high [30]. Further research needs to be conducted to define the additional agronomic characteristics of the germplasm, including yield, resistance, etc., allowing for the germplasm to be purified, so its seeds can be used for rapid popularization. In recent years, the demand for CIF has rapidly increased alongside increasing sales of Ganmaoling granules; however, wild CIF resources are constantly decreasing because of over-exploitation and extreme weather [5]. Breeding and promoting elite cultivars to improve the supply and quality of CIF can ensure the continued production and quality of TCMs, decoction pieces, dispensing granules and other products, as well as protect *C*. *indicum* germplasm resources.

Yellow is the main color of CIF, which is mainly composed of carotenoids, largely lutein [31]. These carotenoids are synthesized through the methyl-erythritol phosphate (MEP) pathway. Flavonoids including linarin are synthesized through the shikimate pathway. Therefore, the synthesis of carotenoids and flavonoids do not have direct correlation. Our results also showed that there was no significant correlation between flavonoids (linarin, luteolin, and apigenin) and b* (yellow-blue chromaticity). However, b* was positive with phenolic acids (neochlorogenic acid, chlorogenic acid, and isochlorogenic acids B and C). The correlation phenomenon between the yellow and phenolic acids of CIF, apart from linarin, luteolin and apigenin of CIF need to be further studied. Furthermore, although CIF drying methods such as drying in the sun or drying after steaming are recommended by the *Chinese Pharmacopoeia 2020*, other drying methods, including stoving, stoving after stir-fried blanching and stoving after steam blanching, are widely used in CIF production. Different processing methods will lead to different colors of CIF [14]. The influences of the drying methods on the content of the bioactive constituents of CIF remain to be studied.

## Conclusions

In our study, we established a new UPLC method to determine nine major bioactive constituents of CIF; furthermore, we revealed the correlations between the nine bioactive constituents and CIF color parameters. Our results showed that the concentrations of CIF’s neochlorogenic acid, chlorogenic acid, isochlorogenic acids A, B and C, and acacetin, as well as the comprehensive quality *γ*_i_ of CIF, were positively correlated with and could be estimated based on color parameters L* and/or b*. However, there were no significant correlations between the color parameters and CIF’s chemical marker—linarin. Our work largely contributes to quality evaluations of CIF by color to breed and procure high-quality medicinal materials.

## Supporting information

S1 Fig Linearity assessment of the nine reference standards under UPLC conditions. (TIF)

S2 Fig Cluster gragh of the 70 C. indicum germplasms using SPSS. (TIF)

S1 Table Precision of CIF sample constituents in each measurement under UPLC conditions. (DOCX)

S2 Table Stability of the nine constituents under UPLC conditions. (DOCX)

S3 Table Recoveries of the nine constituents under UPLC conditions. (DOCX)
