# Supplementary figures and images for: Evaluation of Chrysanthemi Indici Flos germplasms based on nine bioactive constituents and color parameters
Source: PLoS One. 2023 Apr 21;18(4):e0283498. doi: 10.1371/journal.pone.0283498 (PMC10121038; doi:10.1371/journal.pone.0283498)

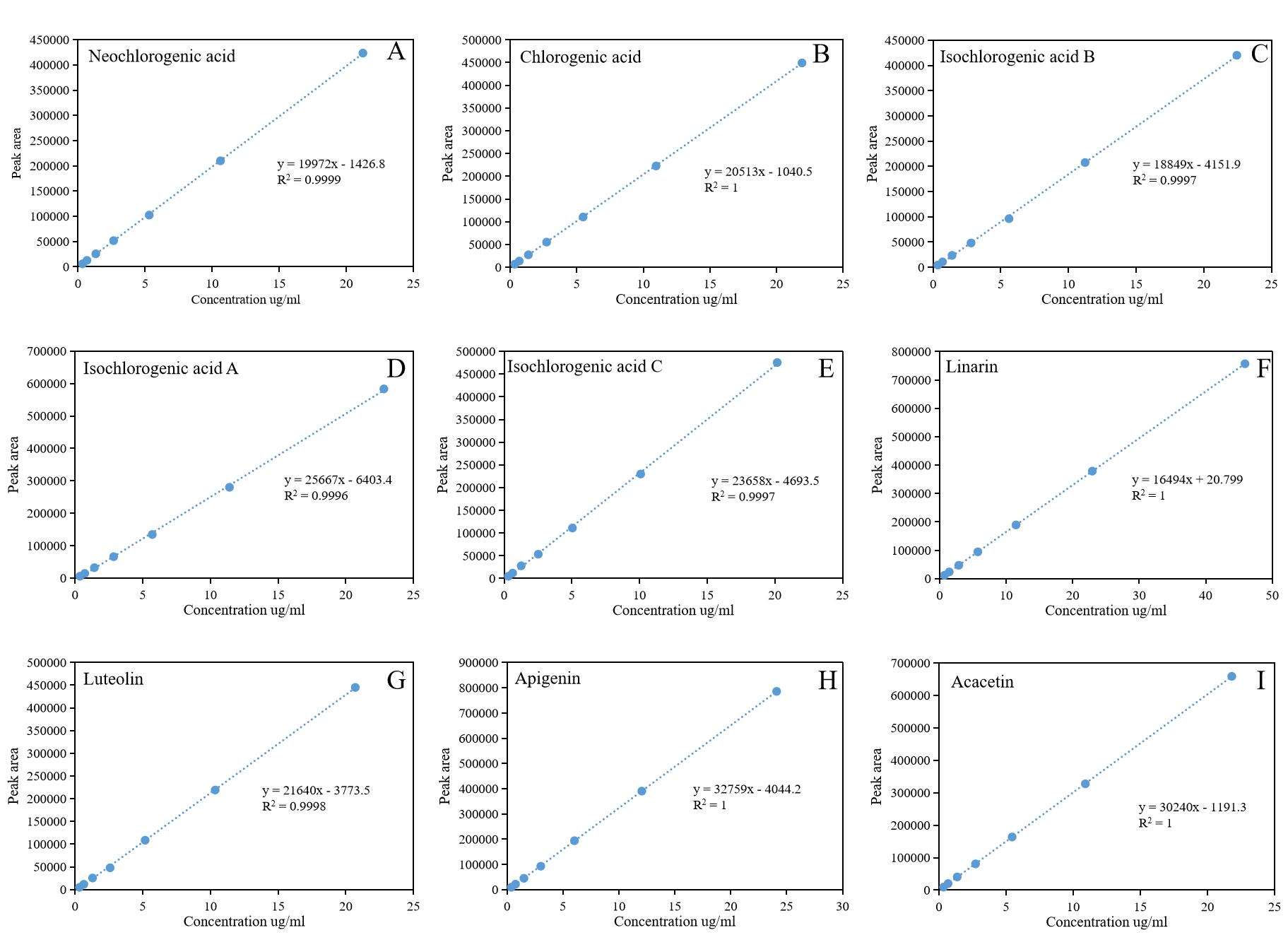

Supplement: S1 Fig — (TIF) [file pone.0283498.s001.tif]

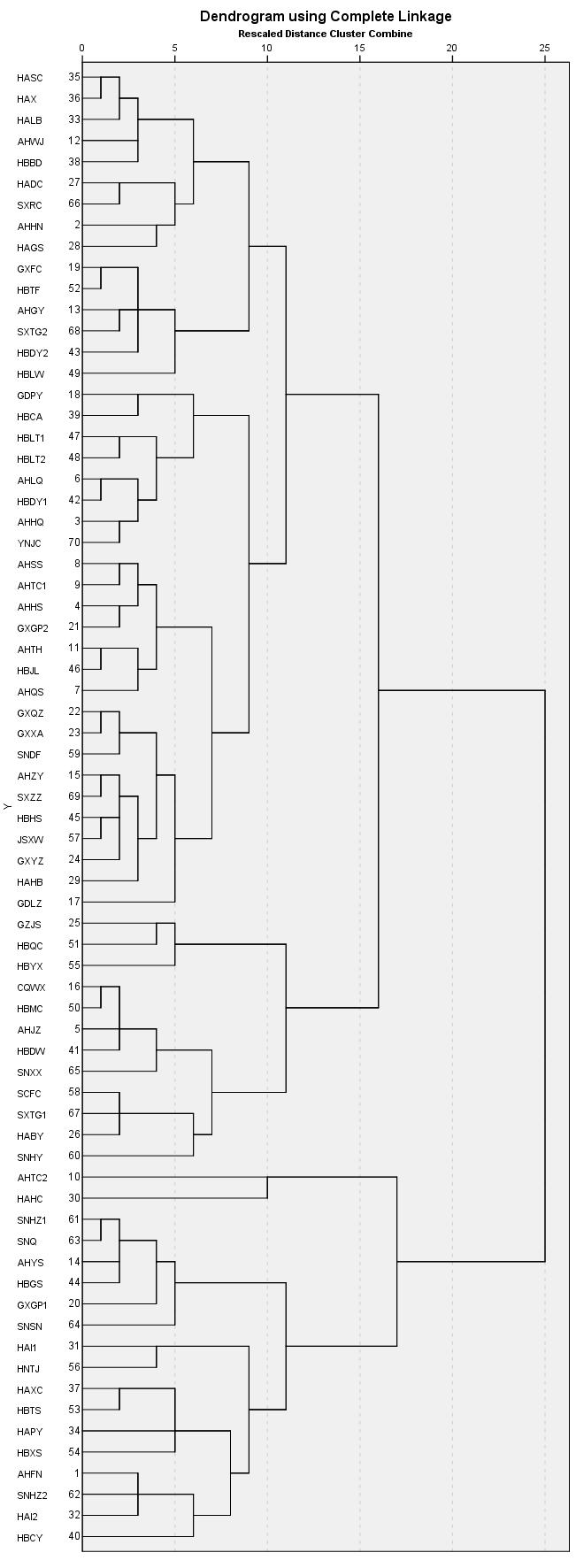

Supplement: S2 Fig — (TIF) [file pone.0283498.s002.tif]
