# Supplementary material for: Evaluation of Chrysanthemi Indici Flos germplasms based on nine bioactive constituents and color parameters
Source: PLoS One. 2023 Apr 21;18(4):e0283498. doi: 10.1371/journal.pone.0283498 (PMC10121038; doi:10.1371/journal.pone.0283498)
Supplement: S1 Table — (DOCX) [file pone.0283498.s003.docx]

**S1 Table.** **Precision of CIF sample constituents in each measurement under UPLC conditions.**

| **Bioactive constituents** | **Concentration in each measurement (mg·g^-1^)** | | | | | | **Average concentration**  **(mg·g^-1^)** | **RSD**  **（%）** |
| --- | --- | --- | --- | --- | --- | --- | --- | --- |
|  | **1** | **2** | **3** | **4** | **5** | **6** |  |  |
| Neochlorogenic acid | 0.1707 | 0.1718 | 0.1713 | 0.1696 | 0.1693 | 0.1704 | 0.1705 | 0.58 |
| Chlorogenic acid | 1.1593 | 1.1587 | 1.1547 | 1.1523 | 1.1367 | 1.1478 | 1.1516 | 0.74 |
| Isochlorogenic acid B | 0.1668 | 0.168 | 0.1664 | 0.1677 | 0.1656 | 0.1709 | 0.1676 | 1.20 |
| Isochlorogenic acid A | 2.1604 | 2.1632 | 2.1393 | 2.1465 | 2.097 | 2.1253 | 2.1386 | 1.20 |
| Isochlorogenic acid C | 0.6602 | 0.6603 | 0.6459 | 0.6498 | 0.6325 | 0.6529 | 0.6503 | 1.70 |
| Linarin | 19.9545 | 19.9686 | 20.0015 | 19.923 | 19.8371 | 19.9039 | 19.9314 | 0.29 |
| Luteolin | 0.6396 | 0.6288 | 0.6336 | 0.6411 | 0.6317 | 0.6355 | 0.6351 | 0.74 |
| Apigenin | 0.7144 | 0.691 | 0.7043 | 0.7044 | 0.7003 | 0.7102 | 0.7041 | 1.20 |
| Acacetin | 0.3977 | 0.3994 | 0.3941 | 0.4041 | 0.3988 | 0.4038 | 0.3997 | 0.96 |
