# Supplementary material for: Evaluation of Chrysanthemi Indici Flos germplasms based on nine bioactive constituents and color parameters
Source: PLoS One. 2023 Apr 21;18(4):e0283498. doi: 10.1371/journal.pone.0283498 (PMC10121038; doi:10.1371/journal.pone.0283498)
Supplement: S2 Table — (DOCX) [file pone.0283498.s004.docx]

**S2 Table. Stability of the nine constituents under UPLC conditions.**

| **Bioactive constituents** | **Time** | | | | | | | | **RSD**  **（%）** |
| --- | --- | --- | --- | --- | --- | --- | --- | --- | --- |
|  | **0 h** | **2 h** | **4 h** | **8 h** | **16 h** | **24 h** | **32 h** | **48 h** |  |
| Neochlorogenic acid | 8843 | 9068 | 8825 | 8872 | 8819 | 9168 | 9258 | 8814 | 2.00 |
| Chlorogenic acid | 108345 | 107796 | 108667 | 109623 | 109870 | 110569 | 112009 | 111043 | 1.40 |
| Isochlorogenic acid B | 22079 | 22785 | 22425 | 22964 | 21721 | 22286 | 22077 | 22657 | 1.90 |
| Isochlorogenic acid A | 256951 | 257979 | 259100 | 259680 | 260736 | 262888 | 263363 | 260933 | 0.86 |
| Isochlorogenic acid C | 60029 | 60494 | 61108 | 62326 | 60143 | 61304 | 62925 | 63116 | 2.00 |
| Linarin | 1101841 | 1103336 | 1102531 | 1103851 | 1101370 | 1101664 | 1106463 | 1099805 | 0.19 |
| Luteolin | 49741 | 49849 | 49702 | 49951 | 50163 | 50181 | 50492 | 50499 | 0.63 |
| Apigenin | 63023 | 63021 | 63190 | 63741 | 63415 | 63271 | 63537 | 62954 | 0.45 |
| Acacetin | 12159 | 11729 | 11811 | 12044 | 11885 | 12057 | 11839 | 11851 | 1.30 |
