# Supplementary material for: Evaluation of Chrysanthemi Indici Flos germplasms based on nine bioactive constituents and color parameters
Source: PLoS One. 2023 Apr 21;18(4):e0283498. doi: 10.1371/journal.pone.0283498 (PMC10121038; doi:10.1371/journal.pone.0283498)
Supplement: S3 Table — (DOCX) [file pone.0283498.s005.docx]

**S3 Table. Recoveries of the nine constituents under UPLC conditions.**

| **Bioactive constituents** | **Sample weight (g)** | **Active constituent**  **weight of sample (mg)** | **Added weight of**  **reference standards (mg)** | **Detected weight of**  **bioactive constituents (mg)** | **Recovery (%)** | **Average recovery (%)** | **RSD**  **(%)** |
| --- | --- | --- | --- | --- | --- | --- | --- |
| Neochlorogenic acid | 0.12499 | 0.02131 | 0.02132 | 0.04259 | 99.81 | 100.25 | 0.31 |
|  | 0.12498 | 0.02131 | 0.02132 | 0.04267 | 100.20 |  |  |
|  | 0.12499 | 0.02131 | 0.02132 | 0.04273 | 100.40 |  |  |
|  | 0.12500 | 0.02131 | 0.02132 | 0.04275 | 100.6 |  |  |
|  | 0.12499 | 0.02131 | 0.02132 | 0.04262 | 99.96 |  |  |
|  | 0.12498 | 0.02131 | 0.02132 | 0.04273 | 100.50 |  |  |
| Chlorogenic acid | 0.12499 | 0.14394 | 0.14400 | 0.28898 | 100.70 | 99.82 | 0.76 |
|  | 0.12498 | 0.14392 | 0.14400 | 0.28844 | 100.40 |  |  |
|  | 0.12499 | 0.14394 | 0.14400 | 0.28852 | 100.40 |  |  |
|  | 0.12500 | 0.14395 | 0.14400 | 0.28658 | 99.05 |  |  |
|  | 0.12499 | 0.14394 | 0.14400 | 0.28656 | 99.05 |  |  |
|  | 0.12498 | 0.14392 | 0.14400 | 0.28698 | 99.34 |  |  |
| Isochlorogenic acid B | 0.12499 | 0.02094 | 0.02094 | 0.04161 | 98.66 | 98.62 | 0.94 |
|  | 0.12498 | 0.02094 | 0.02094 | 0.04131 | 97.26 |  |  |
|  | 0.12499 | 0.02094 | 0.02094 | 0.04192 | 100.10 |  |  |
|  | 0.12500 | 0.02095 | 0.02094 | 0.04166 | 98.89 |  |  |
|  | 0.12499 | 0.02094 | 0.02094 | 0.04151 | 98.17 |  |  |
|  | 0.12498 | 0.02094 | 0.02094 | 0.0416 | 98.61 |  |  |
| Isochlorogenic acid A | 0.12499 | 0.26730 | 0.26750 | 0.5312 | 98.65 | 99.08 | 0.81 |
|  | 0.12498 | 0.26728 | 0.26750 | 0.53557 | 100.30 |  |  |
|  | 0.12499 | 0.26730 | 0.26750 | 0.53368 | 99.58 |  |  |
|  | 0.12500 | 0.26732 | 0.26750 | 0.53187 | 98.90 |  |  |
|  | 0.12499 | 0.2673 | 0.26750 | 0.53231 | 99.07 |  |  |
|  | 0.12498 | 0.26728 | 0.26750 | 0.52933 | 97.96 |  |  |
| Isochlorogenic acid C | 0.12499 | 0.08128 | 0.08135 | 0.16365 | 101.30 | 99.59 | 1.62 |
|  | 0.12498 | 0.08127 | 0.08135 | 0.16285 | 100.30 |  |  |
|  | 0.12499 | 0.08128 | 0.08135 | 0.16223 | 99.51 |  |  |
|  | 0.12500 | 0.08128 | 0.08135 | 0.16077 | 97.71 |  |  |
|  | 0.12499 | 0.08128 | 0.08135 | 0.1607 | 97.63 |  |  |
|  | 0.12498 | 0.08127 | 0.08135 | 0.16353 | 101.10 |  |  |
| Linarin | 0.12499 | 2.49123 | 2.49150 | 4.96798 | 99.41 | 99.84 | 0.45 |
|  | 0.12498 | 2.49103 | 2.49150 | 4.96366 | 99.24 |  |  |
|  | 0.12499 | 2.49123 | 2.49150 | 4.99328 | 100.40 |  |  |
|  | 0.12500 | 2.49143 | 2.49150 | 4.98748 | 100.20 |  |  |
|  | 0.12499 | 2.49123 | 2.49150 | 4.98135 | 99.94 |  |  |
|  | 0.12498 | 2.49103 | 2.49150 | 4.97796 | 99.82 |  |  |
| Luteolin | 0.12499 | 0.07938 | 0.07935 | 0.16017 | 101.80 | 101.41 | 1.42 |
|  | 0.12498 | 0.07937 | 0.07935 | 0.16065 | 102.40 |  |  |
|  | 0.12499 | 0.07938 | 0.07935 | 0.16061 | 102.40 |  |  |
|  | 0.12500 | 0.07938 | 0.07935 | 0.15806 | 99.16 |  |  |
|  | 0.12499 | 0.07938 | 0.07935 | 0.16081 | 102.60 |  |  |
|  | 0.12498 | 0.07937 | 0.07935 | 0.15877 | 100.10 |  |  |
| Apigenin | 0.12499 | 0.08800 | 0.08810 | 0.17714 | 101.20 | 100.74 | 1.22 |
|  | 0.12498 | 0.08800 | 0.08810 | 0.17659 | 100.60 |  |  |
|  | 0.12499 | 0.08800 | 0.08810 | 0.17749 | 101.60 |  |  |
|  | 0.12500 | 0.08801 | 0.08810 | 0.1767 | 100.70 |  |  |
|  | 0.12499 | 0.08800 | 0.08810 | 0.17774 | 101.90 |  |  |
|  | 0.12498 | 0.08800 | 0.08810 | 0.17472 | 98.44 |  |  |
| Acacetin | 0.12499 | 0.04995 | 0.04995 | 0.10072 | 101.60 | 101.57 | 1.69 |
|  | 0.12498 | 0.04995 | 0.04995 | 0.10179 | 103.80 |  |  |
|  | 0.12499 | 0.04995 | 0.04995 | 0.10109 | 102.40 |  |  |
|  | 0.12500 | 0.04996 | 0.04995 | 0.09922 | 98.62 |  |  |
|  | 0.12499 | 0.04995 | 0.04995 | 0.10086 | 101.90 |  |  |
|  | 0.12498 | 0.04995 | 0.04995 | 0.10043 | 101.10 |  |  |
